# Lower leg muscle structure and function are altered in long-distance runners with medial tibial stress syndrome: a case control study

**DOI:** 10.1186/s13047-021-00485-5

**Published:** 2021-07-07

**Authors:** Joshua Mattock, Julie R. Steele, Karen J. Mickle

**Affiliations:** 1 Biomechanics Research Laboratory, University of Wollongong, NSW Wollongong, Australia; 2 School of Allied Health, Human Services and Sport, La Trobe University, VIC Melbourne, Australia

**Keywords:** Shin splints, Injury prevention, Muscle strength, Muscle structure, Ultrasound, Running

## Abstract

**Background:**

Medial tibial stress syndrome (MTSS) is a common lower leg injury experienced by runners. Although numerous risk factors are reported in the literature, many are non-modifiable and management of the injury remains difficult. Lower leg muscle structure and function are modifiable characteristics that influence tibial loading during foot-ground contact. Therefore, this study aimed to determine whether long-distance runners with MTSS displayed differences in *in vivo* lower leg muscle structure and function than matched asymptomatic runners.

**Methods:**

Lower leg structure was assessed using ultrasound and a measure of lower leg circumference to quantify muscle cross-sectional area, thickness and lean lower leg girth. Lower leg function was assessed using a hand-held dynamometer to quantify maximal voluntary isometric contraction strength and a single leg heel raise protocol was used to measure ankle plantar flexor endurance. Outcome variables were compared between the limbs of long-distance runners suffering MTSS (*n* = 20) and matched asymptomatic controls (*n* = 20). Means, standard deviations, 95 % confidence intervals, mean differences and Cohen’s *d* values were calculated for each variable for the MTSS symptomatic and control limbs.

**Results:**

MTSS symptomatic limbs displayed a significantly smaller flexor hallucis longus cross-sectional area, a smaller soleus thickness but a larger lateral gastrocnemius thickness than the control limbs. However, there was no statistical difference in lean lower leg girth. Compared to the matched control limbs, MTSS symptomatic limbs displayed deficits in maximal voluntary isometric contraction strength of the flexor hallucis longus, soleus, tibialis anterior and peroneal muscles, and reduced ankle plantar flexor endurance capacity.

**Conclusions:**

Differences in lower leg muscle structure and function likely render MTSS symptomatic individuals less able to withstand the negative tibial bending moment generated during midstance, potentially contributing to the development of MTSS. The clinical implications of these findings suggest that rehabilitation protocols for MTSS symptomatic individuals should aim to improve strength of the flexor hallucis longus, soleus, tibialis anterior and peroneal muscles along with ankle plantar flexor endurance. However, the cross-sectional study design prevents us determining whether between group differences were a cause or effect of MTSS. Therefore, future prospective studies are required to substantiate the study findings.

## Introduction

Medial tibial stress syndrome (MTSS), more commonly known as shin splints, is one of the most common forms of exercise induced lower leg pain [1]. Researchers have previously identified several risk factors associated with MTSS development, including increased navicular drop, increased body mass index (BMI), fewer years of running experience, a history of MTSS, female sex and lean lower leg girth [2, 3]. Although numerous risk factors for MTSS have been proposed, managing MTSS remains difficult, with clinicians lacking high-quality evidence for any treatment intervention being better than rest [4].

Two risk factors for MTSS that could easily be modified is structure and function of the lower leg muscles that contribute to the changes in lower leg girth seen in individuals who develop MTSS. Authors have previously reported that compared to asymptomatic controls, MTSS symptomatic individuals displayed less lean lower leg girth [5], reduced ankle plantar flexor endurance capacity [6] and adaptations in lower leg muscle force production [7, 8]. Burne et al. [5] established a causal relationship between reduced lean lower leg girth and MTSS development in males and hypothesised that the amount of lower leg muscle bulk would influence the ability of the lower leg to attenuate ground reaction forces generated at foot-ground contact and ultimately contribute to MTSS development.

Lean lower leg girth is a circumferential measure of lower leg muscle bulk measured while a participant is standing and then corrected for adipose tissue thickness. Lean lower leg girth, however, does not provide information regarding how individual lower leg muscle composition affects the overall circumference measurement. To date, no study could be located that has assessed *in vivo* lower leg muscle cross-sectional area (CSA) or thickness to determine whether reduced lean lower leg girth associated with MTSS is due to atrophy of specific lower leg muscles or a uniform reduction in lower leg muscle size. A better understanding of how lower leg muscle composition differs between MTSS symptomatic and asymptomatic controls will provide evidence to develop future intervention studies that target specific lower leg muscles to treat or prevent MTSS.

The ability of the lower leg to attenuate the ground reaction forces generated at foot-ground contact during running is better explained by the functional capacity of the lower leg muscles [9] rather than its structural composition. Therefore, to determine whether a functional deficit is associated with any structural change to a muscle, muscle strength and endurance metrics should be assessed in combination with *in vivo* structural composition measurements. Researchers have previously reported that when matched to asymptomatic controls, MTSS symptomatic individuals display less ankle plantar flexor endurance, increased flexor hallucis longus plantar flexion torque and a greater isokinetic evertor strength compared to invertor muscle strength [6–8]. However, variability in the characteristics of the MTSS-control matched participant groups within these studies could explain these study results. For example, Madeley et al. [6] did not report participant training volume but acknowledged a reduced sporting activity involvement of the MTSS symptomatic group and the two participant groups assessed by Yuksel et al. [7] were significantly different in age of beginning sporting activity (*p* < 0.056) and training volume (*p* < 0.001). Additionally, to avoid the confounding variable of pain, Saeki et al. [8] matched individuals with a history of MTSS, excluding individuals with current symptoms, to asymptomatic individuals. Consequently, these results should be interpreted with caution because differences between participant groups in training volume or factors involved in managing MTSS, such as rest and muscle atrophy, could have affected the study results.

Given the limitations of previous research described above, there is a need to investigate differences in lower leg muscle function and structure *in vivo* in an MTSS cohort that is well matched to asymptomatic individuals to inform future MTSS treatment studies. Long-distance runners, who continue to train and compete despite suffering MTSS symptoms, present a unique cohort to determine whether individuals with MTSS display differences in lower leg muscle structure or function than matched asymptomatic runners without the results being confounded by the effects of muscle atrophy associated with rest. Therefore, this study aimed to determine whether active long-distance runners with MTSS displayed differences in *in vivo* lower leg muscle structure and function compared to asymptomatic long-distance runners. It was hypothesised that the lower leg muscle structure of the symptomatic limbs of runners with MTSS, characterised by lean lower leg girth, lower leg muscle CSA and thickness, would be compromised compared to limbs of asymptomatic long-distance runners. Furthermore, it was hypothesised that runners with MTSS would display less lower leg muscle strength and less ankle plantar flexor endurance capacity in their symptomatic limbs compared to limbs of asymptomatic long-distance runners.

## Methods

### Participants

A case-control study design was used to assess whether long-distance runners suffering MTSS and abstaining from rest displayed differences in lower leg muscle structure and function compared to matched asymptomatic long-distance runners. Ethical clearance for the study was obtained from the University of Wollongong Human Research Ethics Committee (2015/012) and before inclusion, participants provided informed consent. Participants were included in the study if they were aged over 18 years, ran an average of 30 km per week for no less than 6 months, or were training for a long-distance event of at least a half marathon. From a larger cohort of 64 long-distance runners, eleven MTSS symptomatic individuals were matched on sex, age, height, body mass, weekly running training distance and limb dominance with 11 asymptomatic controls (see Table 1). Nine of the 11 symptomatic individuals experienced bilateral MTSS symptoms (average duration 14.8 ± 9.8 months), providing a total of 20 symptomatic and 20 control limbs.

**Table 1:** Characteristics of the MTSS symptomatic (*n* = 11) and matched control participants (*n* = 11)

Variables	MTSS symptomatic		Control
Sex (female/male)		8/3^a^	8/3^a^
Age (years)		32.9 ± 9.2	32.6 ± 8.9
Height (cm)		1.72 ± 5.1	1.71 ± 8.8
Mass (kg)		68.3 ± 6.3	67.1 ± 8.6
Body mass index (kg/m^2^)		23.2 ± 2.3	23.1 ± 2.7
Weekly running training distance (km)		32.3 ± 12.9	34.1 ± 9.4

All values mean ± standard deviation except for ^a,^ which is a count

The symptomatic participants were confirmed to have MTSS by an experienced podiatrist [JM] based on the diagnostic criteria of Yates & White [10]. Participants were excluded from the study based on the criteria established by Mattock et al. [11].

### Lower leg structure

**Fig. 1:**
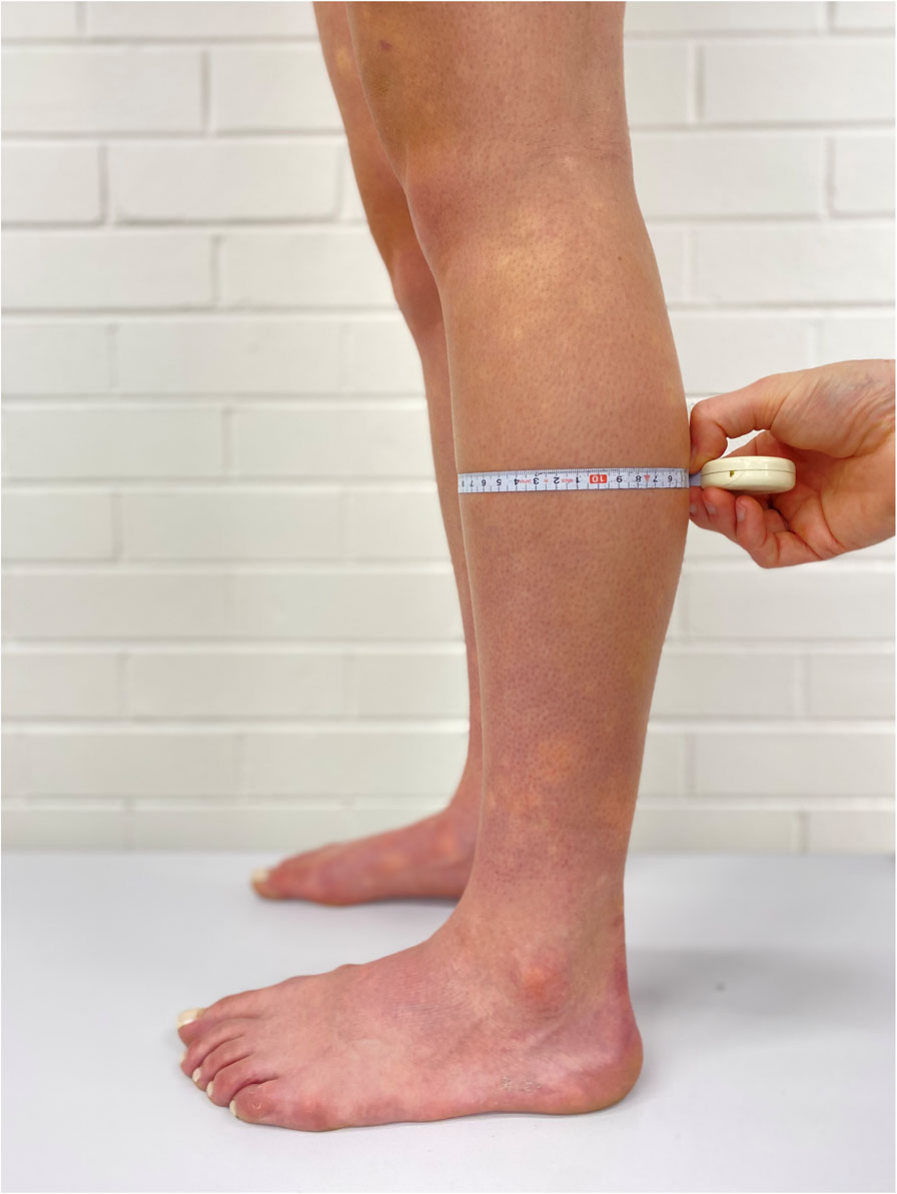
Maximal lower leg girth measurement

Lower leg structure was quantified by assessing each participant’s *in vivo* lower leg muscle thickness and CSA and lean lower leg girth following the procedures described by Mattock et al. [11]. In brief, a Sonosite Edge HD2 ultrasound machine (FUJIFILM SonoSite, Inc., Bothell, WA, USA) was used to assess the thickness of tibialis anterior (TA), the peroneal muscles (P), soleus (SOL), flexor digitorum longus (FDL), flexor hallucis longus (FHL) and medial (GM) and lateral (GL) gastrocnemius. It was only possible to measure the CSA of TA, P, FDL and FHL due to constraints of the ultrasound probe. The FDL, FHL, P and TA were measured following the protocol described by Crofts et al. [12] and the SOL, GM and GL were measured following the protocol described by Weiss et al. [13]. Muscle thickness (mm) and CSA (mm^2^) were measured using Image J software (National Institute for Health, Bethesda, MD, USA). Lean lower leg girth was calculated by measuring the maximal lower leg girth while a participant was standing (see Fig. 1) [5]. This value was then corrected for adipose tissue thickness, which was measured from the GM ultrasound image, and then normalised to lower leg length using Eq. 1.

1 $$\frac{maximal\;lower\;leg\;girth-adipose\;tissue\;thickness}{lower\;leg\;length}$$

### Lower leg function

Lower leg function was represented by measuring each participant’s lower leg maximal voluntary isometric contraction (MVIC) strength and lower leg ankle plantar flexor endurance capacity as described by Mattock et al. [11]. In brief, lower leg MVIC strength was assessed by the participants performing a series of 3–5 s maximal efforts against a hand-held dynamometer (Gollehon, Lafayette Inc., IN, USA) for the TA, P, SOL, FDL, FHL and gastrocnemius. The strength measurements were then normalised to each participant’s body weight (% BW). Ankle plantar flexor endurance was assessed using a single leg heel raise protocol with participants attempting to perform as many single leg heel raises as possible [11].

### Reliability

Before collecting data, the chief investigator [JM] measured the lower leg muscle thickness and CSA, lean lower leg girth and lower leg MVIC strength of a convenience sample of two females and four males on two separate occasions. Intraclass correlation coefficients for lower leg muscle thickness (all > 0.671) and CSA (all > 0.932), lean lower leg girth (0.992) and lower leg MVIC strength (all > 0.776) confirmed the measurements were moderate to highly reliable. The ankle plantar flexor endurance protocol has previously been shown to have excellent test-retest reliability [6].

### Statistical analysis

Descriptive statistics (means, standard deviations, 95 % confidence intervals and mean differences) were calculated for each variable for the MTSS symptomatic and control limbs. Nine of the eleven MTSS symptomatic participants experienced bilateral symptoms. Therefore, a mixed-model linear regression design was used to determine whether there were any significant (*p* < 0.05) differences in the outcome variables between the 20 MTSS symptomatic and 20 matched control limbs. The effect size was calculated using Cohen’s *d* where 0.2, 0.5 and 0.8 were considered small, moderate and large, respectively [14]. The outcome variables included lower leg muscle thickness and CSA, lean lower leg girth, lower leg MVIC strength and lower leg ankle plantar flexor endurance capacity. All statistical tests were performed using SPSS 26.0 for Windows (IBM Inc., Armonk, NY, USA).

## Results

### Lower leg structure

Descriptive statistics for the lower leg muscle thickness and CSA and lean lower leg girth for the MTSS symptomatic and control limbs are shown in Table 2. The MTSS symptomatic limbs displayed a moderate to large effect size for a significantly smaller FHL CSA, a smaller SOL thickness but a larger GL thickness than the control limbs. Despite structural differences in some of the lower leg muscles, lean lower leg girth did not significantly differ between the MTSS symptomatic and control limbs.

**Table 2:** Lower leg muscle structure for 20 MTSS symptomatic and 20 control limbs

Variables	MTSS symptomatic limbs	95 % CI	Control limbs	95 % CI	Mean difference	Cohen’s *d*	*p*-value
FDL thickness (mm)	15.9 (2.8)	14.7–17.0	15.9 (2.2)	14.8–17.1	0	0	0.951
FDL CSA (mm^2^)	243.3 (88.9)	200.4-286.5	277 (96.1)	234.1-320.1	33.7	0.36	0.252
FHL thickness (mm)	18.9 (2.5)	17.3–20.7	20.6 (4.0)	18.7–22.3	1.7	0.51	0.056
FHL CSA (mm^2^)	481.9 (122.3)	404.9-550.1	538.6 (126.0)	461.4-605.8	56.7	0.46	0.042*
GL thickness (mm)	15.8 (2.1)	14.9–16.7	13.9 (1.9)	13.0-14.8	-1.9	-0.95	0.007*
GM thickness (mm)	20.3 (3.6)	18.9–21.7	18.9 (2.1)	17.5–20.3	-1.4	-0.47	0.114
P thickness (mm)	15.6 (2.4)	14.6–16.7	14.9 (2.0)	13.8–15.9	-0.7	-0.32	0.288
P CSA (mm^2^)	377.1 (77.4)	337.4-410.6	354.2 (57.7)	317.0-387.2	-22.9	-0.34	0.273
SOL thickness (mm)	16.5 (2.7)	15.0–18.0	18.4 (3.1)	16.9–19.9	1.9	0.65	0.016*
TA thickness (mm)	25.1 (2.6)	23.3–26.9	24.2 (3.7)	22.4–26.0	-0.9	-0.28	0.289
TA CSA (mm^2^)	672.8 (160.7)	591.6-763.2	686.9 (108.4)	605.1-779.2	14.1	0.10	0.671
Lean lower leg girth (normalised to lower leg length)	0.94 (0.73)	0.91–0.98	0.95 (0.83)	0.91–0.99	0.01	0.01	0.813

Values are mean (± SD), *p < 0.05

*CI *confidence interval, *FDL *flexor digitorum longus, *FHL *flexor hallucis longus, *GL *gastrocnemius lateral head, *GM *gastrocnemius medial head, *P *peroneals, *SOL *soleus, *TA *tibialis anterior, Mean difference = Control – MTSS symptomatic limbs

*significant difference

### Lower limb function

The lower leg MVIC strength and ankle plantar flexor endurance results, grouped by participant limbs, are shown in Table 3. Compared to their control counterparts, the MTSS symptomatic limbs displayed a moderate to large effect size for significant deficits in lower leg MVIC muscle strength of the FHL, P, SOL and TA. Furthermore, a large effect size was found for MTSS symptomatic participants who displayed a statistically significant deficit in ankle plantar flexor endurance capacity, completing on average 56 % fewer heel raises compared to control participants.

**Table 3:** Lower leg muscle function for 20 MTSS symptomatic and 20 control limbs

Variables	MTSS symptomatic limbs	95 % CI	Control limbs	95 % CI	Mean difference	Cohen’s *d*	*p*-value
FDL (% BW)	15.8 (5)	12.8–18.7	18.6 (6.8)	15.6–21.6	2.8	0.47	0.111
FHL (% BW)	20.3 (5.8)	16.1–24.4	27 (11.2)	22.8–31.1	6.7	0.75	0.023*
Gastrocnemius (% BW)	75 (10.0)	68.1–85.0	79.6 (14.1)	72.7–89.6	4.6	0.38	0.085
P (% BW)	27.9 (6.3)	24.5–31.4	33.5 (7.5)	30.1–36.9	5.6	0.81	0.010*
SOL (% BW)	63.7 (13.5)	55.3–71.2	72.7 (15.9)	65.3–80.5	9	0.61	0.035*
TA (% BW)	33.5 (7.8)	28.0-38.8	42 (12.3)	36.5–47.3	8.5	0.83	0.005*
Ankle plantar flexor endurance (heel raise repetitions)	33.2 (12.7)	5.1–66.2	75.4 (73.8)	47.3-108.4	42.2	0.80	0.005*

Values are mean (± SD), *p < 0.05

*CI *confidence interval, *FDL *flexor digitorum longus, *FHL *flexor hallucis longus, *GL *gastrocnemius lateral head, *GM *gastrocnemius medial head, *P *peroneals, *SOL * soleus, *TA *tibialis anterior, Mean difference = Control – MTSS symptomatic limbs

*significant difference

## Discussion

Long-distance runners who continued to train despite suffering MTSS symptoms displayed a smaller FHL CSA, a thinner SOL but a thicker GL, together with strength deficits in the FHL, P, SOL and TA and less ankle plantar flexor endurance. The implications of these differences in muscle structure and function are discussed below.

Of the muscles assessed in the present study, the ankle plantar flexor muscles demonstrated greater structural changes than other lower leg muscles in the long-distance runners suffering MTSS. The MTSS symptomatic limbs displayed less FHL CSA and SOL thickness but greater GL thickness than control limbs. Although not significant, there was a trend for less FHL thickness and greater GM thickness in MTSS symptomatic limbs. Differences identified in muscle thickness and CSA between MTSS symptomatic and control limbs coincided with lower strength output of the associated muscles. That is, the smaller FHL and SOL size were consistent with lower MVIC strength of both muscles. In contrast, although MTSS symptomatic limbs displayed a significantly thicker GL and a trend towards a thicker GM, there was no significant between-group difference in gastrocnemius strength.

Despite differences in individual muscle thickness and CSA, lean lower leg girth did not significantly differ between MTSS symptomatic and control limbs. Although lean lower leg girth is reported to be a risk factor associated with developing MTSS [5], these findings are not supported by case control comparisons [15]. The results of the current study suggest that although SOL thickness and FHL CSA are lower in MTSS symptomatic limbs, these smaller sizes are likely offset by a thicker GL and, to a lesser extent, GM thickness, masking any between group differences in overall lean lower leg girth.

A thinner SOL and lower SOL MVIC strength, combined with a thicker GL, could be compensatory strategies by MTSS symptomatic participants to reduce SOL traction on the tibia. Naderi et al. [16] prospectively assessed SOL muscle activity in 112 active students and concluded that a higher peak SOL electromyography amplitude was associated with MTSS development. Furthermore, Edama et al. [17] assessed lower leg muscular attachments in 100 Japanese cadavers. They concluded that compared to men, women have a significantly greater proportion of SOL attachment (*p* < 0.001) at the middle and distal thirds of the medial margin of the tibia, which coincides with the site of MTSS pain. Given the findings of Naderi et al. [16] and that 73 % of the cohort in this study were women, it is likely that alterations in motor patterning similar to those seen in individuals with lower back pain, resulted in reduced motor unit recruitment and traction of the SOL at the site of pain [18]. Furthermore, we postulate that MTSS symptomatic individuals instead utilised the gastrocnemius muscle to generate ankle plantar flexion torque, ultimately resulting in atrophy and reduced strength of the SOL muscle.

MTSS symptomatic limbs in the current study also displayed a significant deficit in ankle plantar flexor endurance capacity compared to control limbs. This is unsurprising given the significantly lower ankle plantar flexor muscle strength, notably reduced SOL strength, considering its endurance capacity associated with postural tasks [19]. These findings are also consistent with earlier research of Madeley et al. [6] who reported ankle plantar flexor endurance deficits in MTSS symptomatic individuals compared to a reference group. Deficits in ankle plantar flexor endurance have previously been hypothesised to increase forces transferred to the tibia contributing to MTSS development [20]. Phuah et al. [21] reported that the greatest negative tibial bending moment occurs in the sagittal plane during midstance, resulting in peak tensile strain occurring at the posteromedial distal aspect of the tibia and coincides with the site of MTSS pain [21, 22]. Considering the positive tibial bending moment generated in the sagittal plane by the ankle plantar flexor muscles, weaker plantar flexor muscles and smaller ankle plantar flexor endurance capacity could render MTSS symptomatic participants less able to counteract a negative tibial bending moment. The significant deficit in ankle plantar flexor endurance of the MTSS symptomatic limbs and the large effect size represents a clinically significant difference between groups. Whether lower SOL, FHL and P strength is a cause or effect of MTSS remains unclear. It could, however, explain the slow recovery time from MTSS because individuals who attempt to increase their training load would be less able to withstand the negative tibial bending moment. SOL is particularly important in modulating the negative tibial bending moment. In asymptomatic runners, SOL has a substantially larger capacity to produce ankle plantar flexion torque than the gastrocnemius, producing a force of around eight times body weight compared to three times body weight produced by the gastrocnemius [23]. Furthermore, the oblique course of FHL from the distal two-thirds of the posterior surface of the fibula to the base of the distal phalanx of the hallux makes it ideally suited to resist the tensile strain at the posteromedial tibial border [24]. Reduced FHL CSA and MVIC strength would result in less capacity to resist a negative tibial bending moment. Based on these findings we postulate that MTSS symptomatic individuals are less able to withstand negative tibial bending moments and experience greater tibial strains. Current evidence suggests that when assessed prospectively, individuals who develop MTSS do not display differences in ankle plantar flexor strength than control participants [25]. However, no prospective study could be located that assessed the strength of individual lower leg muscles of MTSS symptomatic patients in isolation. Therefore, future prospective studies are required to determine whether individuals who develop MTSS display reduced SOL, FHL and P MVIC muscle strength over time.

The FHL and TA help control medial longitudinal arch (MLA) height and attenuate ground reaction forces during the stance phase of gait. Naderi et al. [16] concluded that dynamic foot pronation during running was a significant predictor for developing MTSS, hypothesised to increase internal tibial rotation, subsequently imposing higher strains in the tibia [3]. Lower FHL and TA strength in MTSS symptomatic runners could contribute to greater and prolonged lowering of the MLA during stance, contributing to higher and prolonged tibial strains. Furthermore, weakness of the FHL and TA could reduce the ability of the lower leg to attenuate the ground reaction force generated at foot-ground contact, which is hypothesised to increase tibial loading and contribute to MTSS development [5]. The clinically significant differences in FHL and TA strength suggest that rehabilitation protocols should involve FHL and TA strengthening exercises. However, to better understand how lower leg musculature influences dynamic foot pronation and MTSS development, further prospective research is required to assess the strength of the FHL, TA and tibialis posterior to elucidate their possible involvement in MTSS development.

Another possible explanation for the lower strength of FHL, P, SOL and TA in MTSS symptomatic participants could be due to pain and associated neuromuscular adaptations. Although the mechanics are not entirely understood, individuals who experience pain (e.g. lower back pain) display altered muscle activation patterns and morphological muscle changes [18]. Further prospective studies are required to better understand changes to structural and functional muscle characteristics associated with MTSS development without the results being confounded by pain. Although it is difficult to draw strong conclusions from these data, the findings of this study could be generalised to other running populations at risk of MTSS.

### Limitations

It is acknowledged that, as a cross-sectional study, we cannot determine whether the between group differences identified in this study were a cause or effect of MTSS development. Furthermore, the small sample size prevents definitive conclusions from being made. We were also not able to assess the structure of tibialis posterior due to constraints of our ultrasound probe. Tibialis posterior contributes to ankle plantar flexion and foot inversion and, therefore, assists in reducing the negative tibial bending moment during the stance phase of gait. Future research should be prospective in design and assess the structure and function of tibialis posterior to determine its role in resisting negative tibial bending moments.

## Conclusions

Compared to well-matched control participants, runners suffering MTSS symptoms displayed less FHL CSA and SOL thickness but greater GL thickness, as well as lower FHL, SOL, P and TA MVIC strength and ankle plantar flexor endurance capacity. These differences may contribute to the slow recovery time typically seen by MTSS patients because they might be less able to withstand the negative tibial bending moment generated during midstance, which can cause greater tibial strains. Furthermore, future investigation of asymptomatic runners using a prospective design is required to determine whether the between group differences identified in this study are consistent in runners who develop MTSS.

## Data Availability

Data are available on request from Joshua Mattock (jmattock@uow.edu.au) for inclusion in future meta-analyses. Sharing of individual data is not available because this was not included in the participant consent.

## Abbreviations

BMI: Body mass index
CSA: Cross-sectional area
FDL: Flexor digitorum longus
FHL: Flexor hallucis longus
GL: Lateral gastrocnemius
GM: Medial gastrocnemius
MLA: Medial longitudinal arch
MTSS: Medial tibial stress syndrome
MVIC: Maximal voluntary isometric contraction
P: Peroneal muscles
SOL: Soleus
TA: Tibialis anterior
